# Contact rate and risk factors of classical swine fever disease in commercial and smallholder pig farms, Karanganyar, Central Java, Indonesia

**DOI:** 10.14202/vetworld.2021.758-763

**Published:** 2021-03-24

**Authors:** Rama Dharmawan, Bambang Sumiarto, Hendra Wibawa, Ira Pramastuti, Sutiyarmo Sutiyarmo, Bagoes Poermadjaja

**Affiliations:** 1 Department of Veterinary Public Health, Faculty of Veterinary Medicine, Gadjah Mada University, Yogyakarta, Indonesia; 2 Disease Investigation Center Wates, Yogyakarta 55651, Indonesia; 3 Department Fisheries and Animal Husbandry, Karanganyar 57712, Indonesia

**Keywords:** classical swine fever, contact rate, odds ratio, off-farms, on-farms, risk factors

## Abstract

**Background and Aim:**

Classical swine fever (CSF) is one of the primary diseases in animals in Indonesia, particularly areas that supply pig meat to the country, such as Karanganyar district, Central Java. The government has tried to prevent and control the disease by vaccination, but it has not yet given effective results. Therefore, another attempt to prevent the recurrence of CSF cases is to apply biosecurity in pig farms by looking for risk factors associated with on-farm and off-farm contact. This study aims to determine the contact rate and investigate the risk factors associated with on-farm and off-farm contact in commercial and smallholder pig farms in Karanganyar, Central Java, Indonesia, in the context of controlling CSF disease.

**Materials and Methods:**

This study used a cross-sectional study design in which the pig farm was designed as the observed epidemiological unit. The contact structure data were conducted by sampling using a two-stage random method. We selected Karanganyar district because it is the center of a pig farm in the Central Java Province and has many CSF cases in several years before. The study was conducted for more or less 1 month from August to September 2019. The contact data were collected from 37 smallholder farms and 27 commercial farms within interviews. Risk factors for contact with pigs were analyzed using logistic regression using theStatistix Program version 8.0.(www.statistix.com)

**Results:**

In comparison to smallholder farms, commercial farms had 2.38 and 3.32 times higher contact rate in outside farms and inside farms, respectively. Two factors increased the risk for on-farm contacts including commercials type farm (p=0.0012; odds ratio [OR]=8.32) with contact rate of 1.24 times/day and the time interval of CSF vaccination for 1-3 months (p=0.0013; OR=8.43) with contact rate of 0.98 times/day, and three factors increased the risk for off-farm contacts including the commercial farm type (p=0.012; OR=4.88) with 1.50 contact/day, the time interval of CSF vaccination for 1-3 months (p=0.036; OR=3.83) with 1.30 contact/day, and farmers with experience in pig husbandry <5 years (p=0.075; OR=3.56) with 1.13 contact/day.

**Conclusion:**

This study shows that commercial farms and short CSF vaccination intervals increased the risk of either off-farm or on-farm contacts. The contact structure of pig farms in Karanganyar district is similar to that in other areas in Indonesia. Reducing the risk of contacts either outside or inside the pig farms is essential to prevent disease transmission. Enhancing communication and education to pig farmers and surveillance is also necessary to prevent such diseases in pigs.

## Introduction

Classical swine fever (CSF), astrategic pig animal disease -influence significant economic losses because of its high morbidity and mortality rates [1]. The Indonesian government has made efforts to control and eradicate CSF by increasing the understanding of epidemiological risk factors of CSF. According to the principles of veterinary epidemiology, an animal disease does not occur randomly in the general population, but it occurs in clusters at particular times and locations with specific disease patterns [2]. Identification of disease patterns and investigation of risk factors, such as biosecurity status, direct and indirect transmission, and vaccination, are likely to reduce disease incidence in a population [3].

Several studies have discussed risk factors, including the application of livestock biosecurity, patterns of disease transmission, and failures of vaccination, that may contribute to the transmission of pathogenic swine diseases, especially CSF, in Indonesia. By contrast, contact is defined as anything that came in and out of a farm that may have contacted animals or their products outside or inside the farm. Persons who have such contacts included workers, technical service, livestock owners, stable technicians, vaccinators, and traders. Objects with such contacts include livestock and equipment such as ropes, pens for carrying animals, footwear, and other things such as feed and vehicles. For example, a previous study identified risk factor analysis for the transmission of CSF in West Timor, Indonesia [4].

Reducing the risk for animal contacts on and off a farm is a part of biosecurity management. If the risk factors for animal contact on the farm can be calculated, disease control strategies can be implemented to reduce or prevent disease transmission. Contact risk can be assessed by counting the number of visiting contacts (people, animals, and goods) on and off the farm. However, few studies have identified risk factors involved in persons or objects with contacts or the number of contacts in a pig farm.

Based on the purpose and scale of production, Indonesia’s pig farms are generally categorized as commercial and smallholder farms. Types of pig farms between regions in Indonesia differ slightly. The smallholder pig farms in East Nusa Tenggara (NTT) generally raise pigs as a secondary source of income. The breeders keep at least one pig in a simple bamboo cage. The feed comes from local agricultural products, sometimes usingswill. Furthermore, because of a lack of knowledge about animal health and poor biosecurity practices, the breedersrarely report to the livestock service officials when a pig is sick or dead [5]. By contrast, the commercial farms in NTT typically have a livestock population of more than 200, use commercial feed and an automatic drinking water system, clean the cages regularly, perform artificial insemination, and maintain a record of the livestock [6]. However, their biosecurity practices are generally still low [7].

By contrast, the type of pig farming is determined based on local government regulations in Central Java, especially the Karanganyar Regency [8]. Farms with more than 150 animals are categorized as commercial farms, whereas farms with 150 or fewer animals are classified as traditional or smallholder farms. The pigs are raised on commercial and smallholder farms with comparable water sources, farm locations, and levels of cleanliness. The two types of farms differ in biosecurity practices, feed sources, barrier wall covering, livestock trading, livestock recording, and vaccination.

There aremany studies on the causes of CSF transmission in pig farms [1,3,5], but there hasn’t been research on the contact risk factors and the number of animal contacts that occur outside of pig farms (off-farm) or inside of pig farms(on-farm) in Indonesia. This study aims to determine off-farm and on-farm contact rates in pig farms and risk factors associated with contacts that potentially affect CSF infection in commercial and smallholder pig farms in Karanganyar, Central Java, Indonesia. As mathematical modeling is useful for understanding and assessing the epidemiological dynamics of the disease, and used to understand the animal contact risk factors in this study [9].

## Materials and Methods

### Ethical approval

Ethical approval for this study was obtained from the Animal Ethics Committee of the Faculty of Veterinary Medicine, Gadjah Mada University, Yogyakarta, with the registration number 0089/EC-FKH /Ex/2019, dated July 30, 2019.

### Study design, period, and area

**Figure-1:**
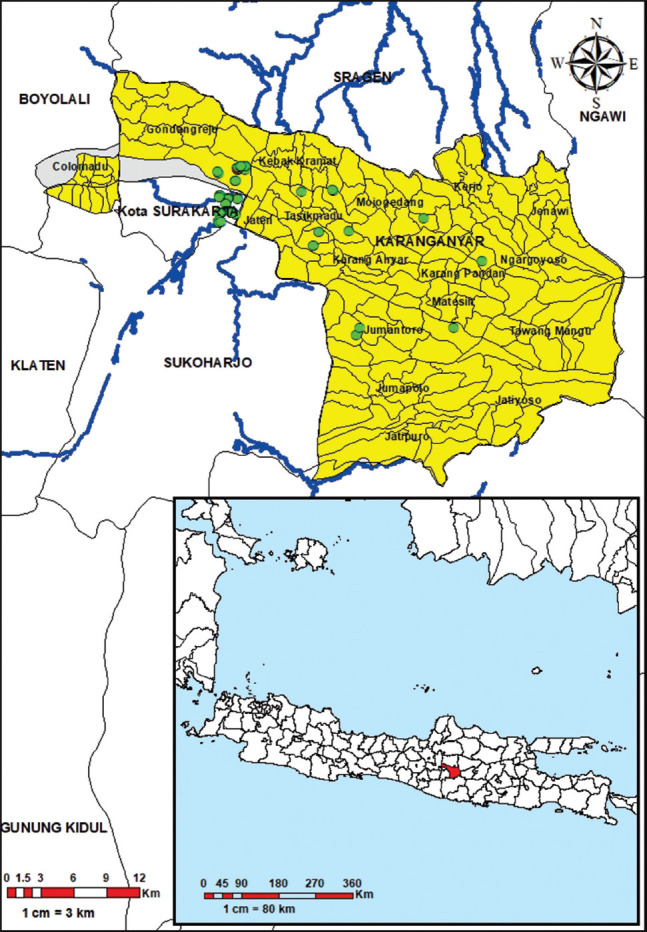
Location of pig farms (green dots): A cross-sectional study was conducted in Karanganyar district, Central Java, Indonesia. Several farms close to each other; hence, they are appeared to have the same green dots (ArcGIS 10.8 (ESRI)) .

The authors conducted cross-sectional study in the Karanganyar district from September to December 2019. The farmers profiling process was carried out from September to October 2019, and farmers monitoring was carried out from November to December 2019. The study area is described through a spatial map in Figure-1.

### Sample and data collection

The farms were selected using a two-stage random method. Data were collected from 64 farms, consisting of 37 smallholder farms and 27 commercial farms. Data on the contact structure and risk factors were obtained through interviews with farmers and from workers using a questionnaire. The mean period of recording contact structure data was 30.23 days. Before the study began, we collected the basic information for farm profiling. Then, we visited the farms again to distribute logbooks for recording contact data. The farmers were taught how to fill in a notebook, and they were asked to make an entry every day for approximately a month. Furthermore, livestock service officers and researchers visited farmers at least three times in Karanganyar district to ensure that farmer records data are correct to reduce information bias during data recording.

### Definition of contact

Contact was defined as anything that came in and out of the farm that might have contact with pigs or their products outside the farm (“off-farm contact”) or inside the farm (“on-farm contact”). These contacts involved people, such as workers, technical service personnel, livestock owners, stable technicians, vaccinators, and traders. These contacts also involved objects such as livestock and equipment such as ropes, pens for carrying pigs, and other things such as footwear, feed, and vehicles.

### Statistical analysis

Farm profiling data and logbook questionnaires containing daily information on on-farm and off-farm contacts were compiled and organized in an MS Excel file and underwent statistical analysis in Statistix version 8.0 [10]. The average number of contacts/day (contact rate) was determined by the number of off-farm or on-farm contacts in each type of farm divided by the mean of the days of observation (30.23). A logistic regression model was used to analyze the relationship between the categorical risk factors and contacts [11,12]. On-farm and off-farm risk factors were used as independent variables, and the average number of contacts was categorized as a dependent variable.

## Results

### Farm profile and the number ofpigs contacts

We collected the contact structure data from 64 pig farms during the study period, which comprised 27 commercial farms (42%) and 37 smallholder farms (58%). The farms were profiled, and their numbers of on-farm and off-farm contacts were determined (Table-1). We found that the commercial farms had higher off-farm and on-farm contact rates, at 1.50 and 1.24 contacts/day, respectively than those of the smallholder farms at 0.63 and 0.82 contacts/day, respectively. Approximately 67% (43/64) of the pig farms were located near residential areas, and they had a higher off-farm contact rate at 1.16 contacts/day than the 33% (21/64) of the farms that were located far away from the residential area (0.67 contacts/day). By contrast, the farms near residential areas had a lower on-farm contact rate at 0.97 contacts/day than that of farms far from residential areas, at 1.05 contacts/day.

**Table-1:** The proportion of farm according to observed variables and their respective contact rates (average contact per day).

Variable	Category	Number of farms	Number of farms (%)	Off-farm		On-farm	
	
				Contact rate/day	Contact rate/week	Contact rate/day	Contact rate/week
Livestock model	1) Commercial	27	42	1.50	10.47	1.24	8.65
(Liv_Mod)	2) Smallholder	37	58	0.63	4,41	0,82	5.77
Close to residential area (Clo_res)	1) Yes	43	67	1.16	8.11	0.97	6.82
	2) No	21	33	0,67	4.68	1.05	7.38
Length of vaccination	1) 1-3 months	30	47	1.30	9.13	0,98	6.88
(Len_Vac)	2) 3-6 months	7	11	1.16	8.13	1,22	8,54
	3) 6-12 months	12	19	0.90	6.31	0,80	5,63
	4) >12 months	15	23	0.40	2.78	0,67	4,67
Experience (Liv_Exp)	1) <5 years	16	25	1.13	7,90	1,14	7,99
	2) >5 years	48	75	0.96	6.70	0.95	6.67
Workers stay on the farm (Wo_Sta_Fa)	1) Yes	14	22	1.50	10.52	0.91	6.36
	2) No	50	78	0.86	6.01	1.03	7.18

The pigs received CSF vaccination regimes with four different intervals: 47% (39/64) were vaccinated with an interval of 1-3 months, 11% (7/64) with an interval of 3-6 months, 19% (12/64) with an interval of 6-12 months, and 23% (15/64) with an interval of more than 12 months.

Thefarms vaccinated in an interval of 1-3 months were more likely to have more off-farm contact, at 1.30 contacts/day, than those in an interval of 3-6 months, at 1.16 contacts/day; 6-12 months, at 0.90 contacts/day; and more than 12 months, at 0.40 contacts/day. By contrast, the farms with the vaccination interval of more than 12 monthshad a lower on-farm contact rate than those with an interval of 1-3 months, at 0.98 contacts/day; 3-6 months, at 1.22 contacts/day; and 6-12 months, at 0.80 contacts/day.

The farmers with <5 years of experience in pig farming had higher off-farm and on-farm contact rates, at 1.13 and 1.14 contacts/day, respectively than those of farmers with more than 5 years of experience, at 0.96 and 0.95 contacts/day, respectively. By contrast, the pig farms whose workers lived inside farms had higher off-farm contact rates but lower on-farm contact rates, at 1.50 and 0.91 contacts/day, respectively, than those with workers living outside the farm, at 0.86 and 1.03 on-farm contacts/day, respectively.

### Risk factors

Several factors, including the commercial farm model (Liv_Mod 2) (ß = 1.58470; *P* = 0.012; odds ratio [OR]=4.88), vaccination with an interval of 1-3 months (Len_Vac 1) (ß=1.34324; p=0.036; OR=3.83), and farmers with pig husbandry experience of fewer than 5 years (Liv_Exp 1) (ß=1.27025; p=0.075; OR=3.56), were identified to increase the risk of off-farm contact (Table-2).

**Table-2:** Analysis of risk factors off-farms pigs contact in Karanganyar district.

Predictor variable	ß	SE	Coef./SE	P	OR	95% CI	

						Lo	Up
Constant	−2.52865	0.626	−4.04	0.0001	-	-	-
Liv_Mod2	1.58470	0.629	2.52	0.012	4.88	1.42	16.73
Len_Vac1	1.34324	0.642	2.09	0.036	3.83	1.09	13.49
Liv_Exp1	1.27025	0.714	1.78	0.075	3.56	0.88	14.44

ß=Coefficient, SE=Standard error, P*=P*-value, OR=Odds ratio, CI=Confidence interval

By contrast, only two factors, vaccination with an interval of 1-3 months (Len_Vac 1) (ß= 2.13134; p=0.0013; OR=8.43) and commercial farm model (Liv_Mod 2) (ß=2.11827; p=0.0012; OR=8.32), were identified to increase the risk of on-farm contact (Table-3).

**Table-3:** Analysis of risk factors on-farms pig contact in the Karanganyar district.

Predictor variable	ß	SE	Coef./SE	P	OR	95% CI	

						Lo	Up
Constant	−2.20968	0.590	−3.74	0.0002	-	-	-
Len_Vac1	2.13134	0.656	3.25	0.0013	8.43	2.33	30.48
Liv_Mod2	2.11827	0.659	3.21	0.0012	8.32	2.28	30.29

ß=Coefficient, SE=Standard error, P*=P*-value, OR=Odds ratio, CI=Confidence interval

## Discussion

### Off-farm contact rate and risk factors

The risk factors for off-farm contact are strongly associated with the commercial farm model (p=0.0012) and significantly increase the risk of off-farm contact (OR=4.88, 95% CI: 1.42-16.73) compared to the smallholder farm model.

The commercial farms with large livestock populations tend to have many workers who live near the farms. Some workers could have animals at home, and they sometimes also work as pig traders. Consequently, the off-farm contact rates in commercial farms were 2.38 times higher, at 1.50 contacts/day or 10.47 contacts/week, than those of the smallholder farms, at 0.63 contacts/day or 4.41 contacts/week. In addition, farmworkers might have more contacts because they could visit other farms or deliver pigs to the slaughterhouse.

On- and off-farm activities without good biosecurity will risk introducing disease agents [13], such as pork sales that occur at least once a month on commercial farms. Traders generally enter the pig pen area to select and collect pigs. They typically visit more than 1 farm location at a time. The pig traders’ entry into and exit from the farm cause risk of disease transmission [14]. A commercial farm in East Nusa Tenggara (ENT) has similar conditions to those in Karanganyar. Commercial farms in ENT are at significant risk of contracting the disease because there is contact outside the farm. Commercial farms are generally located near other pig farms. Pig carcasses are only disposed of in the trash; pigs are slaughtered; and vehicles that enter the farm are rarely disinfected [7].

Risk factors for off-farm contact during the vaccination regime with an interval of 1-3 months have a strong association (p=0.036). The vaccination interval of 1-3 months increases contact risk (ß=1.34324), with the OR of 3.83 fold (95% CI: 1.09-13.49) compared to other risk factors. The officers or veterinarians providing vaccine, the officials performing artificial insemination, and pig traders are at risk of transmitting pathogenic diseases through their contaminated clothing [13,15]. A one time vaccination to pigs aged 7-8 weeks is sufficient for areas with no CSF cases. By contrast, high-risk or endemic areas should vaccinate the pigs twice, first at 5 weeks and 5 weeks later, with a booster shot [16].

By contrast, the farmers with experience <5 years were found to have a strong association (p=0.075) with off-farm contact; their off-farm contact rate was higher than those of farmers with more than 5 years of experience, with the OR of 3.56 fold (95% CI: 0.88-14.44). These data indicate that novice farmers could have more risk contact outside a farm. Farming experience is related to the proper handling of animals and appropriate management of workers, waste, and guests; thus, it affects disease risk [3,17]. Farmers who visit each other without knowing their health status will be at risk ofcontracting the disease. Thus, farming experience is essential in controlling illnesses that enter a farm and treating diseases.

### On-farm contact rate and risk factors

The vaccination interval of 1-3 months is significantly associated with on-farm contact (p=0.0013) and increases the risk of contact (b=2.13134) for livestock, with an increased OR by 8.43 fold (95% CI: 2.33-30.48) compared to other risk factors. Pig farms that performed CSF vaccinations with a short interval increased the contact of the farmers or farm workers with pigs. The magnitude of the application of commercial farm vaccination 55% (15/27) was higher than that of smallholder farms 40% (15/37) (Table-1) because commercial farms had pregnant sows and a starter more frequently.

In the Karanganyar district, the farms purchased the vaccines together, and farmers dispensed them. This arrangement likely affects disease transmission due to the staff’s movement from one farm to another. This situation is different from that in East Nusa Tenggara, where vaccination is generally performed by officials or local government officers [17]. Similar to what was observed for off-farm contact, the commercial farms had a strong association (p=0.0012) with on-farm contact (ß=2.11827) and a significantly higher OR (OR=8.32; 95% CI: 2.28-30.29) than those of smallholder farms. These results indicate that the commercial farms have 3.32 times more contacts inside the farm than those in the smallholder farms. The contact rate in the commercial farms was 1.24 contacts/day or 8.65 contacts/week, whereas the smallholder farms’ rate was 0.82 contacts/day or 5.77 contacts/week. A larger livestock population requires more cage workers and health workers. Thus, the activities of the cage workers, visiting guests, and animal health workers may increase the risk of disease transmission [13,18].

## Conclusion

This study demonstrates that commercial farms and short CSF vaccination intervals increase the risk of off-farm or on-farm contact. Farmers with <5 years of experience in pig farming also increased the off-farm contact rate. The contact structure of pig farms in the Karanganyar district is similar to that in other areas in Indonesia. Therefore, reducing the risk of contacts outside or inside the farm is essential in prevention of disease transmission. Other efforts, such as surveillance, communication, and education of pig farmers, are necessary to prevent CSF in pigs.

## Authors’ Contributions

This research is a part of RD’s original research work for his master program. RD, BS, and HW designed a research work plan. RD drafted the manuscript with support from BS, HW, and BP. RD and SS conducted surveillance and field sampling. RD, HW, and IP did laboratory work. RD, HW, and BS analyzed the results. BP provided laboratory support on sample testing and at the Disease Investigation Center Wates and support for the field study. All authors read and approved the final manuscript.
